# PET Imaging in Preclinical Anti-Aβ Drug Development

**DOI:** 10.1007/s11095-022-03277-z

**Published:** 2022-05-02

**Authors:** Stina Syvänen, Silvio R. Meier, Sahar Roshanbin, Mengfei Xiong, Rebecca Faresjö, Tobias Gustavsson, Gillian Bonvicini, Eva Schlein, Ximena Aguilar, Ulrika Julku, Jonas Eriksson, Dag Sehlin

**Affiliations:** 1 Department of Public Health and Caring Sciences, Uppsala University, Dag Hammarskjöldsväg 20, 75185 Uppsala, Sweden; 2 BioArctic AB, Stockholm, Sweden; 3 Department of Medicinal Chemistry, Uppsala University, Uppsala, Sweden; 4 PET Centre, Uppsala University Hospital, Uppsala, Sweden

**Keywords:** Alzheimer’s disease, amyloid-beta, animal models, drug development, Positron Emission Tomography (PET)

## Abstract

Positron emission tomography (PET), a medical imaging technique allowing for studies of the living human brain, has gained an important role in clinical trials of novel drugs against Alzheimer’s disease (AD). For example, PET data contributed to the conditional approval in 2021 of *aducanumab*, an antibody directed towards amyloid-beta (Aβ) aggregates, by showing a dose-dependent reduction in brain amyloid after treatment. In parallel to clinical studies, preclinical studies in animal models of Aβ pathology may also benefit from PET as a tool to detect target engagement and treatment effects of anti-Aβ drug candidates. PET is associated with a high level of translatability between species as similar, non-invasive protocols allow for longitudinal rather than cross-sectional studies and can be used both in a preclinical and clinical setting. This review focuses on the use of preclinical PET imaging in genetically modified animals that express human Aβ, and its present and potential future role in the development of drugs aimed at reducing brain Aβ levels as a therapeutic strategy to halt disease progression in AD.

## INTRODUCTION

New treatments for peripheral diseases such as cancers, diabetes and cardiovascular disease have improved considerably over the past 20 years. In addition to a better general health status in the population, these new treatments have contributed to an increased life expectancy, not only in the high-income countries but also in developing countries. As aging is the most important risk factor for developing Alzheimer’s disease (AD), i.e. the most common form of dementia, the number of people suffering from AD will dramatically increase during the next decades (1). Unfortunately, the development of novel treatments for AD has not been as successful as drug development for other major lethal diseases such as those mentioned above. A recent glimmer of hope in the quest for treatment is the conditional approval of antibody *aducanumab* that targets amyloid-beta (Aβ) in the AD brain (2). This was the first new approved drug for AD since 2003 when memantine, a symptomatic rather than disease modifying treatment, was introduced (3, 4).

**Fig. 1:**
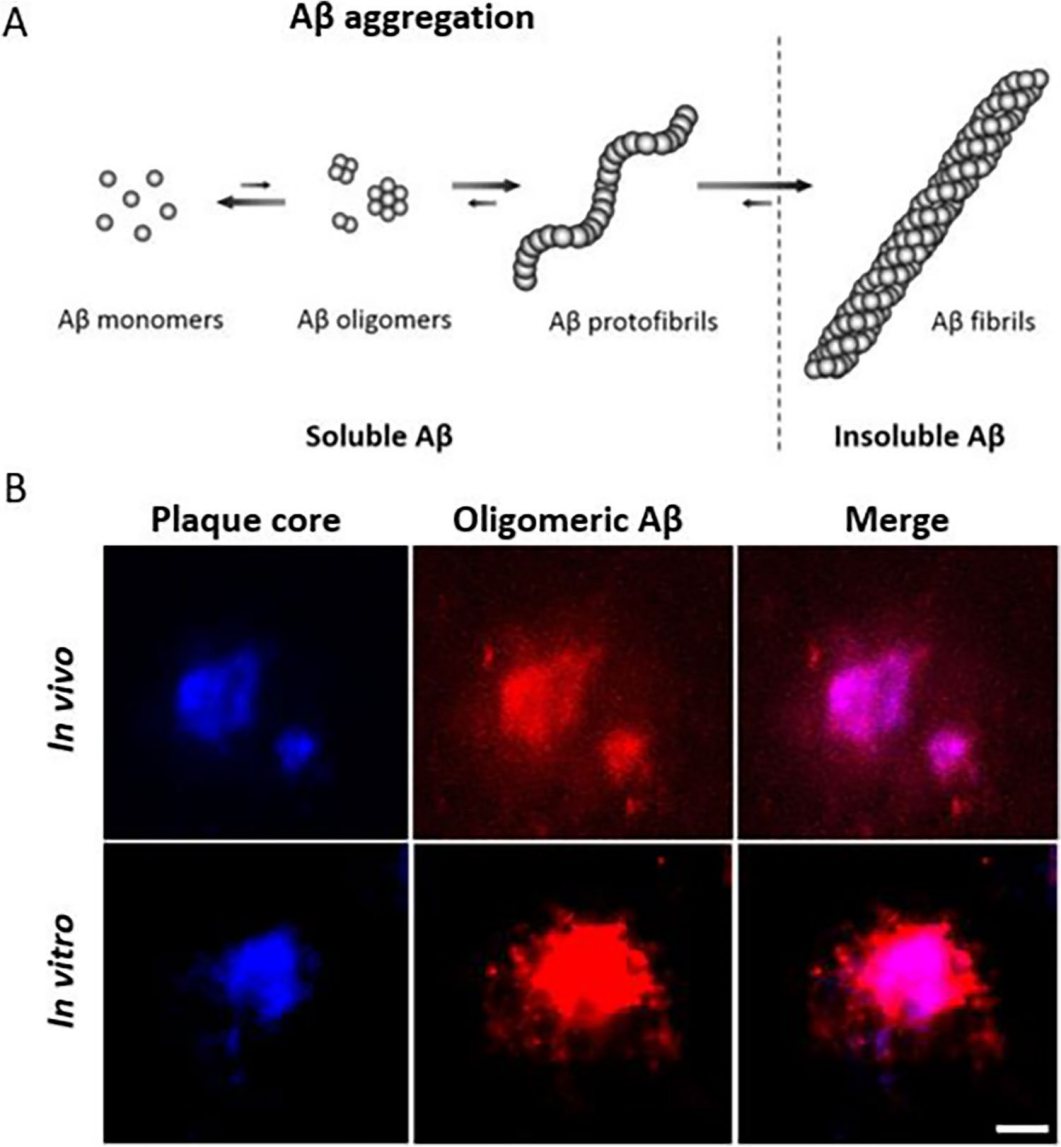
Aggregation of Aβ. (**A**) The Aβ peptide misfolds and aggregates into larger protein assemblies. Aβ fibrils are insoluble and may be deposited as plaques. (**B**) Plaques are protein assemblies, sometimes with an amyloid core. Oligomeric Aβ is present in the dense core of amyloid plaques and in a halo surrounding the core. Upper row: NAB61 antibody conjugated to Alexa Fluor 594 in red applied topically and detected by multiphoton microscopy *in vivo* shows oligomeric Aβ surrounding dense plaques labeled with methoxy XO4 in blue. Lower row: Postmortem *in vitro* staining of dense plaques confirms oligomeric Aβ in an area surrounding the core. Scale bar: 10 μm. Figure (**B**) obtained from Koffie *et al*. 2009 with permission from the publisher (7).

One obvious reason for the difficulties in developing new treatments against AD is the inherent complexity of the disease. Accumulation of brain Aβ, along with neurofibrillary tau tangles and neuroinflammation, accompanied by synaptic loss and neurodegeneration, are important hallmarks of the disease. Measurement of Aβ and tau in the brain or in cerebrospinal fluid (CSF) is also the basis for many of the most frequently used biomarkers for the disease. According to the ‘amyloid cascade hypothesis’, Aβ misfolding, followed by the formation of aggregates of increasing size that are eventually deposited as *amyloid plaques* (Fig. 1)*,* trigger the cascade of pathological changes observed in the AD brain. However, the exact interplay between Aβ and proteins such as tau is not known (5, 6).

Preclinical models of AD are usually characterized by Aβ aggregation, neuroinflammation and in some cases, neuronal degeneration either with or without changes in cognitive behavior. However, tau tangles are usually absent in models of Aβ aggregation. Despite this lack of a link between Aβ and tau, animal models of Aβ pathology are used extensively for preclinical studies of novel drug candidates that act on Aβ. For example, animal models have been important tools to facilitate the development of AD immunotherapy, i.e. treatments based on antibodies, such as *aducanumab* and other therapeutic antibody candidates (2, 8–10).

As AD in most cases is a slowly progressing disease, Aβ pathology and therapeutic intervention should preferably be investigated in longitudinal studies, ideally with repeated measurements of relevant biomarkers. However, few preclinical experimental methods allow for this. Instead, brain tissue (or CSF) is isolated from separate animals at discrete time points after treatment start or onset of disease. Thus, one animal is required for one data point leading to high numbers of experimental animals for any given study. Further, few preclinical methods allow for studies of the entire brain. Instead, methods are based on sampling, e.g. biopsies, sections, fluid samples etc., from the brain tissue. In addition, *post mortem* tissue or samples collected through invasive procedures may not correctly reflect the complex situation in the living brain.

Positron Emission Tomography (PET) is a non-invasive molecular imaging method that can be used to diagnose various neurological diseases including AD and to quantify effects of disease modifying treatments. PET can also be used in the preclinical setting to study the living brain. As such, PET is a true translational method as the same imaging protocols can be applied in experimental animals and humans. PET also allows for repeated measures in one subject. Therefore, preclinical PET does not only reduce the number of animals needed for any given study but may also reduce the variation as each animal can act as its own control. In addition to longitudinal designs and a reduction of animal use, PET allows for examining the whole brain, including investigation of regional differences that are likely to be important for understanding propagation of pathology between brain areas.

PET relies on the administration of minute amounts (nanomoles) of drug-like radiolabelled molecules that are referred to as radioligands or radiotracers, which bind to the target protein under investigation. PET images are quantitative and based on the spatial distribution of the radioactivity that is detected with a PET scanner. The thioflavin-T derived small molecular radioligand [^11^C]PIB, which was first described in the early 2000s (11), binds to the beta-sheet structure of insoluble amyloid plaques. [^11^C]PIB and later developed analogue radioligands are frequently used to diagnose AD and as an inclusion criterion and sometimes as an outcome parameter for clinical studies of Aβ directed therapy (2, 12, 13).

The purpose of the present article is to give the reader an overview of PET imaging in preclinical models of Aβ pathology and to discuss its usefulness and limitations in the development of novel drugs aimed at reducing brain Aβ levels in AD.

### PET Radioligands Used to detEct Aβ Pathology in Preclinical Studies

**Fig. 2:**
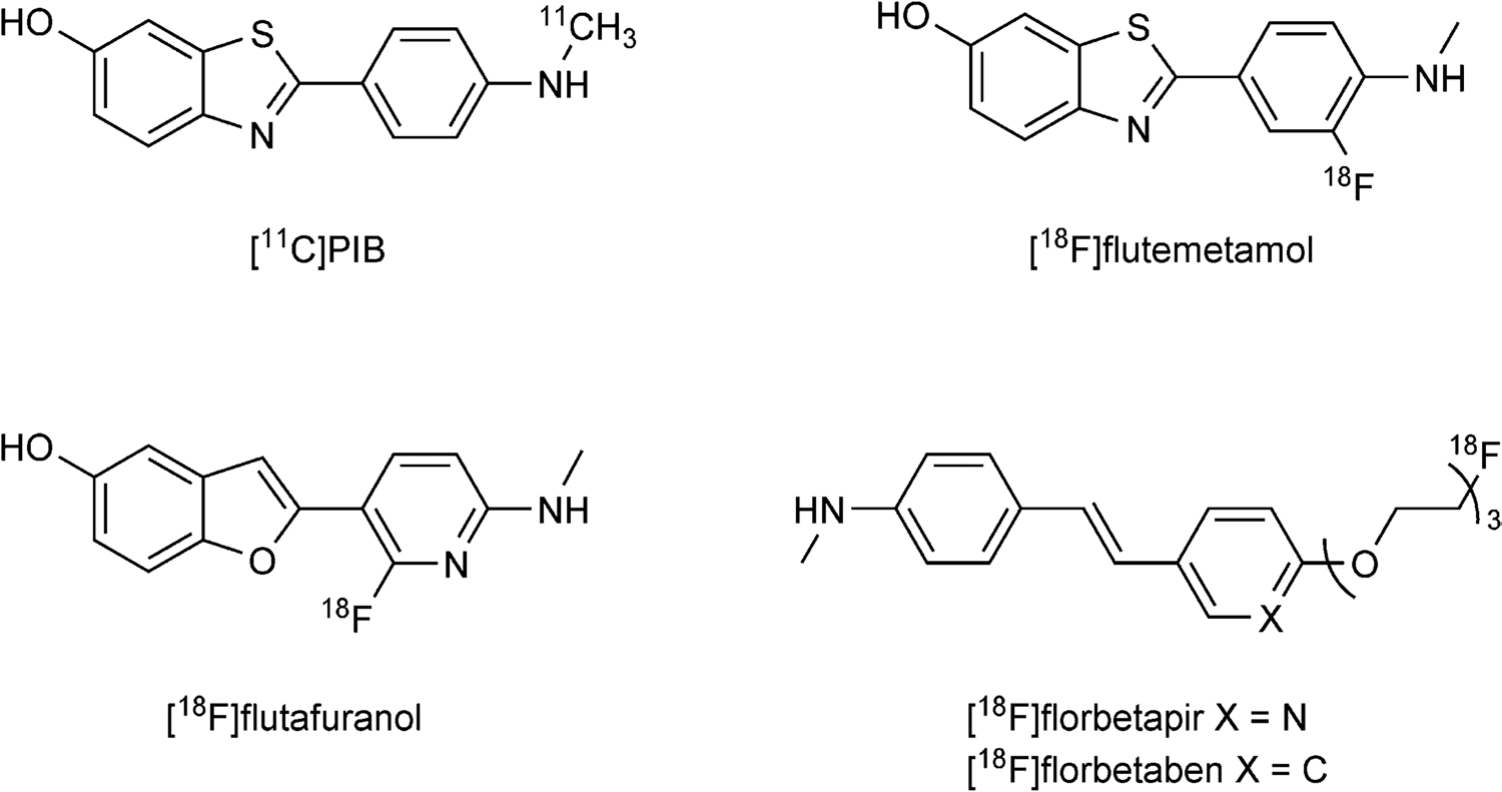
Amyloid PET radioligands. Chemical structures of [^11^C]PIB, the most frequently used ^11^C-labelled radioligand, [^18^F]flutafuranol and three FDA approved ^18^F-labelled radioligands; [^18^F]flutemetamol, [^18^F]florbetapir and [^18^F]florbetaben.

PIB labelled with carbon-11 ([^11^C]PIB), is the gold standard for Aβ imaging with PET. For human application, three fluorine-18 (^18^F) labelled radioligands, [^18^F]flutemetamol, [^18^F]florbetapir and [^18^F]florbetaben (Fig. 2), have been approved by the US Food and Drug Administration (FDA), and as such, they are increasingly used also in preclinical studies. Thus, using ^18^F-labelled amyloid radioligands instead of [^11^C]PIB in preclinical studies is rational from a translational point of view. In addition, the longer half-life of ^18^F (109.8 min) compared to ^11^C (20.4 min) enables scanning of a larger number of animals per radioligand delivery. With access to one preclinical PET scanner, one [^11^C]PIB production batch can be used for scanning one or two individuals while a batch of an ^18^F-amyloid radioligand can be used for up to 10 individuals depending on the protocol used. The experimental cost perspective should not be underestimated since PET is an expensive method, where the cost of one radioligand synthesis may start from 1000 U.S. dollars. Further, ^18^F-radioligands can be shipped to sites that do not have in-house radioligand production.

There are, however, some differences between [^11^C]PIB and the ^18^F-amyloid ligands. The inclusion of fluorine may increase lipophilicity and incidentally some molecules and ^18^F-labelled amyloid ligands tend to distribute into the white matter brain regions more than what is observed with [^11^C]PIB. The increased white matter distribution decreases the differences between specific and non-specific binding, where specific binding is defined as radioligand binding to amyloid while non-specific binding includes off-target binding and unbound radioligand.

Several preclinical studies have compared [^11^C]PIB and ^18^F-labelled ligands. Waldron and co-workers used [^11^C]PIB and [^18^F]florbetaben and found that although intra-group variation between animals was lower with [^18^F]florbetaben, the difference between Aβ-expressing mice and healthy wild-type controls was considerably larger with [^11^C]PIB (Table I) (14). Almost identical results have been seen in human AD patients and healthy controls when comparing these two radioligands (15). In line with these results, comparisons of [^11^C]PIB with [^18^F]florbetapir or with [^18^F]flutemetamol reported better discrimination between Aβ expressing mice and wild-types with [^11^C]PIB (16–18) (Table I).

**Table I:** Difference in Radioligand Binding in Cortex Between Aβ-Expressing Mice and Wild-Type Mice with Amyloid-Radioligands

[^11^C]PIB	[^18^F]florbetapir	[^18^F]flutemetamol	[^18^F]florbetaben	Animal model and age	Reference
21%	14%			5xFAD 11–12 months	Rojas (16)
69%	48%			APPPS1-21 12 months	Waldron (19)
75%		45%		APP23 15–22 months	Snellman (17, 18)*
107%			53%	APPPS1-21 22–25 months	Waldron(14)
70%			10%	*APP*^*NL−G−F*^ /10 months	Meier (20) and Sacher (21) **

*Two different studies, **Two different studies, somewhat different readout (SUV *vs* SUVR)

Antibodies are used extensively in immunohistochemistry to characterize proteins present on tissue sections, including brain sections. In contrast to current amyloid-PET radioligands for which the binding is dependent on the structure of the protein aggregates, antibodies generally bind to specific epitopes based on the amino acid sequences of the protein. Antibodies are large molecules that show very limited and slow distribution to the brain and have therefore not been used for imaging intrabrain targets, although there have been some attempts in the preclinical setting (22–25). Increased interest in immunotherapy of brain disorders has prompted the development of different strategies to increase antibody delivery to the brain, including the use of the transferrin receptor (TfR) as a shuttle for therapeutic proteins and antibodies across the blood–brain barrier (BBB) (26–32). In most cases, a smaller protein moiety that binds to TfR is recombinantly or chemically linked to the therapeutic antibody. In parallel to the development of bispecific therapeutic antibodies that target both TfR and their primary brain target, a few different bispecific antibody-based radioligands engineered to enter the brain via TfR transcytosis have also been described based on either antibody mAb158 (33) or 3D6 (9), which are the murine parent versions of the clinically studied anti-Aβ therapeutics *lecanemab/BAN2401* (13) and *bapineuzumab* (34), respectively. These antibody-based radioligands show a considerably higher specific-to-non-specific signal when compared to [^11^C]PIB, and an ability to detect very low levels of pathology, most likely because they recognize all forms of Aβ aggregates and not only plaques (20, 35–40). Antibody-based PET radioligands have not been translated into clinical use, largely due to their slow systemic pharmacokinetics that require the radioligand to be administered several hours, or days, prior to scanning. Although this may even be an advantage in animal studies as it can increase the number of studied animals per radioligand production as injections and scanning may be performed on separate days, antibody-based neuro PET is challenging for practical reasons in humans. Still, antibodies that can be designed to target specific forms of Aβ aggregates may become important research tools to study Aβ pathology, especially as companion diagnostics to therapeutic anti-Aβ antibodies.

### Quantification of PET Data

PET measures *total* radioactivity in a region of interest. At its simplest, this can be quantified as the measured radioactivity, normalized to the injected dose of radioactivity given as:

1 $$\%\;of\;injected\;dose\;(\%ID/g)\;=\;Radioactivity\;per\;tissue\;weight\;/Injected\;radioactivity\;\times\;100$$

A high bodyweight, and thus a large blood volume, will decrease the concentration of the radioligand in plasma, i.e. the concentration that drives the distribution to the brain. To correct for this and to enable comparison of subjects of different sizes, the outcome measure in PET studies is often the standardized uptake value (SUV) where the measured radioactivity in the region of interest is normalized to the injected radioactivity per bodyweight:

2 $$SUV=Radioactivity\;per\;tissue\;weight\;/\;Injected\;radioactivity\;per\;body\;weight$$

Both %ID/g and SUV reflect the radioactive concentration at the measurement site in relation to the amount of radioactivity injected, but the value in itself does not give any information on specific binding to a target, e.g. amyloid. An outcome parameter that gives a semi-quantitative estimate of target binding is the SUV ratio (SUVR), where the SUV in a region of interest (ROI) is divided by the SUV in a reference region that is devoid of the target. Thus, in AD, this reference region represents radioligand concentrations in pathology free brain tissue. The reference region used often in AD is the cerebellum, or certain parts of the cerebellum, as this region is affected only at late disease stages. In many clinical amyloid-PET studies, the SUVR_ROI/cer_, i.e. the ratio of the SUV in a ROI and the cerebellar SUV, is regarded to indicate “amyloid-positivity” at values above a certain threshold. The threshold value in early PET studies was often set to 1.4, but has, in later studies with more sensitive scanners and larger cohorts of asymptomatic patients and patients in early disease stages, even been set as low as 1.1, meaning that regions that show 10% higher radioligand uptake than cerebellum are regarded as regions with pathological levels of amyloid (41).

The radioligand concentration in the brain tissue changes over time, and thus %ID/g, SUV and SUVR should be reported along with information about the time frame post radioligand administration they represent. Ideally, PET data should be acquired when the specific to non-specific binding ratio is at its maximum. In addition, it is important that a radioligand is eliminated from the blood rapidly as the blood represent 3–5% of the total brain volume, and hence, high radioactivity in the blood may mask the signal from specifically bound radioligand in the brain tissue. PET data in clinical AD studies is often acquired for 60–90 min starting at the time of radioligand administration. However, shorter protocols are also used in which patients are scanned only during 20–40 min, and not necessarily starting at the time of administration but rather when the specific to non-specific signal is high. Short scan times are used to reduce the time that the patient is required to lay still in the scanner, i.e. something that may be difficult for a patient that suffers from a neurological disease. SUV and SUVR reported in animal amyloid-PET studies are often based on data acquired between 30 and 60 min, or between 40 and 60 min, post injection to maximize the specific to non-specific signal.

PET data can also be quantified with more advanced pharmacokinetic modelling to estimate rate constants and binding affinity that are not time dependent. Many of these methods require frequent arterial blood sampling (42). During the development of a new radioligand, it is important that full pharmacokinetic analysis is carried out but the end goal is often to find a simpler method for clinical diagnosis, such as the SUVR. Frequent blood sampling for full pharmacokinetic analysis may not be suitable in clinical settings due to its invasiveness and is not possible in mice due to their limited blood volume. PET-image derived blood concentration curves, obtained from placing a region of interest in the left ventricle of the heart, have been described as an alternative strategy to allow for full pharmacokinetic modeling in preclinical PET studies (43, 44). However, the absolute majority of preclinical PET studies of Aβ pathology have omitted information of blood concentration profiles and relied on semi-quantitative readouts based on reference regions such as the SUVR.

### Molar Activity

One important property of a radioligand is the molar activity, which often is expressed by the unit MBq/nmol or GBq/µmol, i.e. activity per mole compound. Vital for understanding the concept of molar activity is the realization that only a small fraction of all the molecules that are synthesized in a labelling reaction become radioactive. While having identical chemical structures some of the molecules are carrying a radioisotope while the vast majority has the normal abundance of stable isotopes, i.e. ^12^C and ^13^C instead of ^11^C or ^19^F instead of ^18^F. For such radioligands, only about 1 in 1000–2000 molecules carries the radioactive isotope at the end of synthesis. Since the radioactive and non-radioactive molecules have identical chemical structures they express the same pharmacokinetics *in vivo*, and hence, the fate of the labelled compound can be studied by detecting only the radioactive molecules. However, if the target protein is present in very small concentrations and the ratio between radioactive and non-radioactive radioligand molecules is too low, the available binding sites may become occupied with non-radioactive radioligand molecules making imaging of the target impossible. To avoid target saturation or a pharmacological response (i.e. produce a drug-like effect), sufficiently high molar activity is therefore needed.

In the early days of amyloid-imaging, it was concluded that amyloid could not be visualized in transgenic mice (45, 46). These initial preclinical [^11^C]PIB studies were carried out in one of the first developed transgenic Aβ models, the tg-2576 mouse model (47), that did not adequately recapitulate the nature of Aβ plaque pathology seen in the human brain (see also next section). A couple of years later, studies in another transgenic model, APP23 (48), showed that amyloid in the mouse brain could be visualized, but a high molar activity (≈ 200 GBq/µmol) was required to fully reflect the Aβ pathology, while large dense-core plaques were captured also with moderate molar activity (≈ 20 GBq/µmol) (49). Thus, the molar activity will remain an important factor, especially for the detection Aβ pathology characterized by a low number of binding sites, which is likely to be the case for diffuse pathology seen in a subset of patients and some animal models. For [^11^C]PIB and other ^11^C-labelled radioligands, this is a challenge due to the short physical half-life of the radioisotope since the molar activity is directly proportional to the remaining radioactivity of the labelled compound. For example, if it takes 20 min between the end of radioligand synthesis and injection, the molar activity of a ^11^C-based radioligand such as [^11^C]PIB, will have decreased to 50%. Usually, the volume of the administered radioligand is based on the injected radioactivity rather than on the mass, as a certain amount of radioactivity is needed to produce quantitative PET images. Thus, when radioactivity decreases with the physical decay, a larger volume of the radioligand solution will be needed to inject the desired amount of radioactivity. With a larger injection volume, the amount of non-radioactive ligand will increase. The difference seen between [^11^C]PIB and ^18^F-labelled ligands, as discussed in the previous section, could to some degree be a consequence of a larger variation in molar activity for [^11^C]PIB.

### Animal Models of Aβ Pathology

Numerous animal models of Aβ pathology have been described, and detailed information can be found at: www.alzforum.org/research-models. The absolute majority are based on the random insertion of the human amyloid precursor protein (*APP*) gene with known mutations that in humans cause familial AD, usually due to an over-production of Aβ or a shift from Aβ40 to the more aggregation-prone variant Aβ42. The gene is often inserted more than once in the genome, leading to a further increased Aβ generation. While the mutations characterizing the model are always reported, the gene copy number is not, despite the fact that the number of copies will influence the amount of generated Aβ. The previously mentioned tg-2576 model has the human *APP* gene with the Swedish mutation inserted into its genome. Also, APP23 (48), APP_swe_ (50) and Tg-Swe (51) harbor several copies of the human *APP* gene with the Swedish mutation. Other frequently used transgenic models in preclinical AD studies include the 5xFAD (52), 3xTg (53), APPPS1-21 (54), APP_swe_PS1de9 (55) and PS2APP (50) models. As the model names indicate, the inserted human *APP* gene includes several different mutations, while some of the models also harbor mutations in other genes associated with familial AD, e.g. mutations in the presenilin genes (*PS1* and *PS2*). The random insertion of the human gene in transgenic models may cause other unwanted effects on the phenotype of the model. Thus, knock-in models have been generated where the mouse *APP* gene has been humanized, with the addition of various disease causing *APP* mutations. This strategy, although including only one single gene copy, leads to a genetically modified model where the disease causing *APP* gene is located at the correct endogenous site and will therefore achieve natural expression patterns and levels. There are two widely used knock-in models in preclinical AD research: *AppP*^*NL−F*^ and *App*^*NL−G−F*^ (56). Both models harbor the Swedish and the Iberian mutation, while the *App*^*NL−G−F*^ model also includes the Arctic mutation leading to a faster pathology progression. Many of the transgenic models are heterozygous, meaning that the transgene has been inherited only from one transgene parent. Heterozygous breeding is a strategy to avoid detrimental transgene effects, and to slow down pathology progression. Regardless of if the human *APP* gene is heterozygous or homozygous, transgenic animals also express mouse Aβ as the gene is still intact, but mouse Aβ does not seem to aggregate (57). However, knock-in models used in AD research are often homozygous as this doubles the human Aβ production (two alleles instead of one) and the genetic modification is less problematic as the gene is present at its natural endogenous site. Homozygous knock-in mice only express human Aβ. In addition to intrabrain deposits of Aβ, the mouse models also display varying degrees of cerebral amyloid angiopathy (CAA), i.e. deposition of Aβ within blood vessels of the brain, a form of Aβ pathology also present in the human AD brain.

The absolute majority of preclinical PET studies in AD research, and especially studies of new treatments, have been performed in genetically modified mice. However, recently, also some PET studies performed in rat models have been reported (58, 59). In line with mouse models, the rat models include the human *APP* gene with mutations known from familial AD. For example, the McGiIll-R-Thys-APP model that harbors the Swedish and Indiana *APP* mutations (60) and the TgF344-AD model that harbors the Swedish *APP* mutation along with an exon 9 deletion in the presenilin 1 gene (61) have been used in preclinical PET studies to characterize Aβ pathology progression and neuroinflammation (58, 62–64).

### PET Imaging of Aβ Pathology in Rodent Models

The possibility to image Aβ pathology *in vivo* is important for increasing knowledge on how Aβ aggregation proceeds over time and how aggregation influences other disease-related processes in the brain such as neuroinflammation, metabolism and neurodegeneration, i.e. loss of synapses. PET imaging in animals allows for multiple scans using similar protocols as those used in clinical PET studies which increases the translational strength compared to studies where isolation of brain tissue is carried out at a single time-point for subsequent post-mortem analysis of pathology. Further, scanning in humans may be limited to a few scans due to dosimetry and radiation safety, and thus, animal experiments may allow for investigation of several aspects (many radioligands) in one single animal.

Preclinical PET in models of Aβ pathology have also been used in the development of novel radioligands to validate their binding *in vivo*, although it should be acknowledged that many of the amyloid-PET radioligands used clinically today were approved with very limited preclinical work. Limited preclinical validation prior to clinical use is also true for radioligands established to study tau-pathology and synaptic density in human AD (65–68). In the case of the first generation of PET radioligands for tau, extensive off-target binding, mainly to monoamine oxidase B, was found after clinical introduction (69). As a result, the validity of a large number of early clinical PET studies of tau pathology can be questioned. Furthermore, it can be speculated that more thorough preclinical validation could have prevented this early hurdle in developing PET radioligands for tau.

The available PET radioligands visualize “amyloid” which is not the same as Aβ. Amyloids are per definition protein aggregates of fibrillary morphology that form dense beta-sheet structures. Aβ is one such protein, but there are also many other proteins that form amyloid, and these proteins can therefore also be visualized by amyloid-radioligands. For example, alpha-synuclein that forms protein aggregates in the Parkinson’s disease brain and transthyretin that causes amyloidosis in peripheral organs are detected by amyloid radioligands (70, 71).

**Fig. 3:**
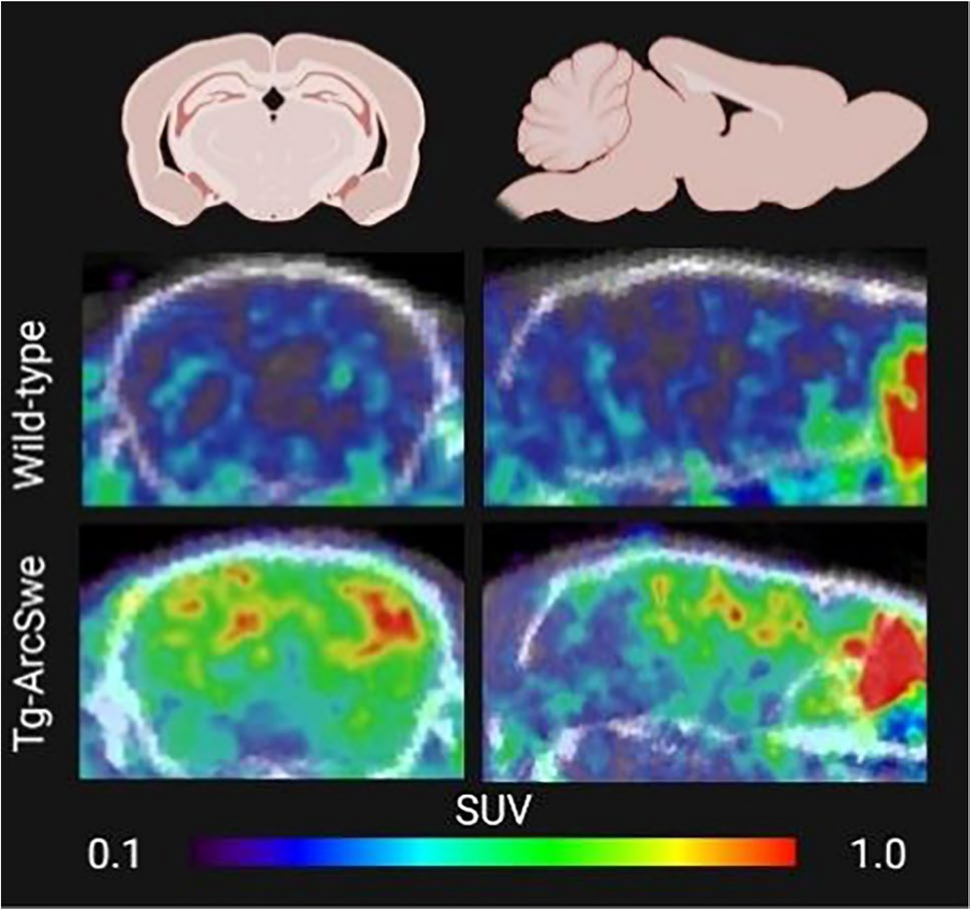
Preclinical amyloid PET. Amyloid imaging with [^11^C]PIB in a wild-type (upper row) and a tg-ArcSwe mouse (lower row). Mice were 18 months old and images represent PET data acquired between 30 and 60 min post radioligand injection.

In AD, the formation of plaques starts by Aβ misfolding, and the subsequent aggregation of misfolded Aβ generates larger protein assemblies that eventually are deposited as insoluble plaques consisting of fibrillary Aβ. It has been shown that the core of amyloid plaques, which contains the majority of binding sites for amyloid radioligands, is surrounded by a halo consisting of more diffuse Aβ assemblies (Fig. 1B) (7). Some familial forms of AD are characterized by dominating diffuse Aβ pathology where the dense-core plaques are less abundant or absent, and consequently, these subjects are sometimes amyloid-negative or display only weak binding of amyloid-radioligands despite very high concentrations of Aβ in the brain (72, 73). The same is observed in many mouse models of Aβ pathology. Snellman and co-workers compared [^11^C]PIB imaging in three models; tg-2576 and APP23 with the Swedish mutation and APP_swe_PS1de9 with the Swedish mutation in combination with a presenilin 1 mutation (17). Only old, 18–21 months, APP23 mice showed a positive [^11^C]PIB signal defined as a SUV ratio of cortex and cerebellum (SUVR_ctx/cer_) above 1. Tg-2576 mice exhibited lower pathology levels in general which could explain the low [^11^C]PIB signal, and confirms the initial studies by Toyama *et al*. in this model (46). When brain sections were analyzed by immunohistochemistry, Aβ pathology in APP_swe_PS1de9 mice exceeded that seen in APP23 mice. Nevertheless, APP_swe_PS1de9 mice showed [^11^C]PIB uptake similar to wild-type mice. Further investigations showed that the Aβ aggregates in APP23 mice were large and Thioflavin S (ThS, fluorescent dye used in histology to stain amyloids) positive, while the aggregates found in the APP_swe_PS1de9 brain were much smaller and did not overlap with ThS staining despite the high number of aggregates. It is known that presenilin mutations shift the relative formation of Aβ40 to Aβ42 (74). Thus, Aβ40 may be required for the generation of large dense-core plaques that can be visualized by amyloid-PET (75). It should be noted that another study using a homozygous version of a similar model based on the Swedish and presenilin 1 mutations did report a positive PET signal in 21 month old mice (76). In theory, homozygous mice should express double the amount of Aβ compared with heterozygous mice. PET SUVR_ctx/cer_ > 1 with [^11^C]PIB have also been reported in 12 and 18 month old tg-ArcSwe mice (Fig. 3) that express the Arctic and the Swedish mutations and in tg-Swe mice at 18 months (35, 36). Another study compared the amyloid radioligand [^18^F]florbetaben in four animal models; APP_swe_/PS2 (Swedish *APP* mutation and a presenilin 2 mutation), APP_swe_/PS1G38A4 (Swedish *APP* mutation and G38A4 presenilin 1 mutation), APP_swe_PS1de9 and APP_Swe_ (77). Only the first model, APP_swe_/PS2, showed SUVR_ctx/cer_ above 1, indicating amyloid positivity at ages 16 and 19 months. The study also showed that SUVR_ctx/cer_ started to decrease in APP_swe_PS1de9 and APP_Swe_ mice at an older age due to emerging pathology in the cerebellum. In line with this, a [^18^F]florbetapir study showed that the difference in SUVR between APPPS1-21 and wild-type mice decreased at an older age (78). Another interesting finding in Aβ models is the appearance of asymmetric plaque burden (79, 80). This characteristic could contribute to the inter-animal variation reported in many studies. However, the ability of PET to analyze pathology in the whole brain should decrease the impact of non-representative sampling of brain tissue which could be the case when only thin sections or discrete tissue samples from a single hemisphere is analyzed, standard in *post mortem* analysis of the brain.

**Fig. 4:**
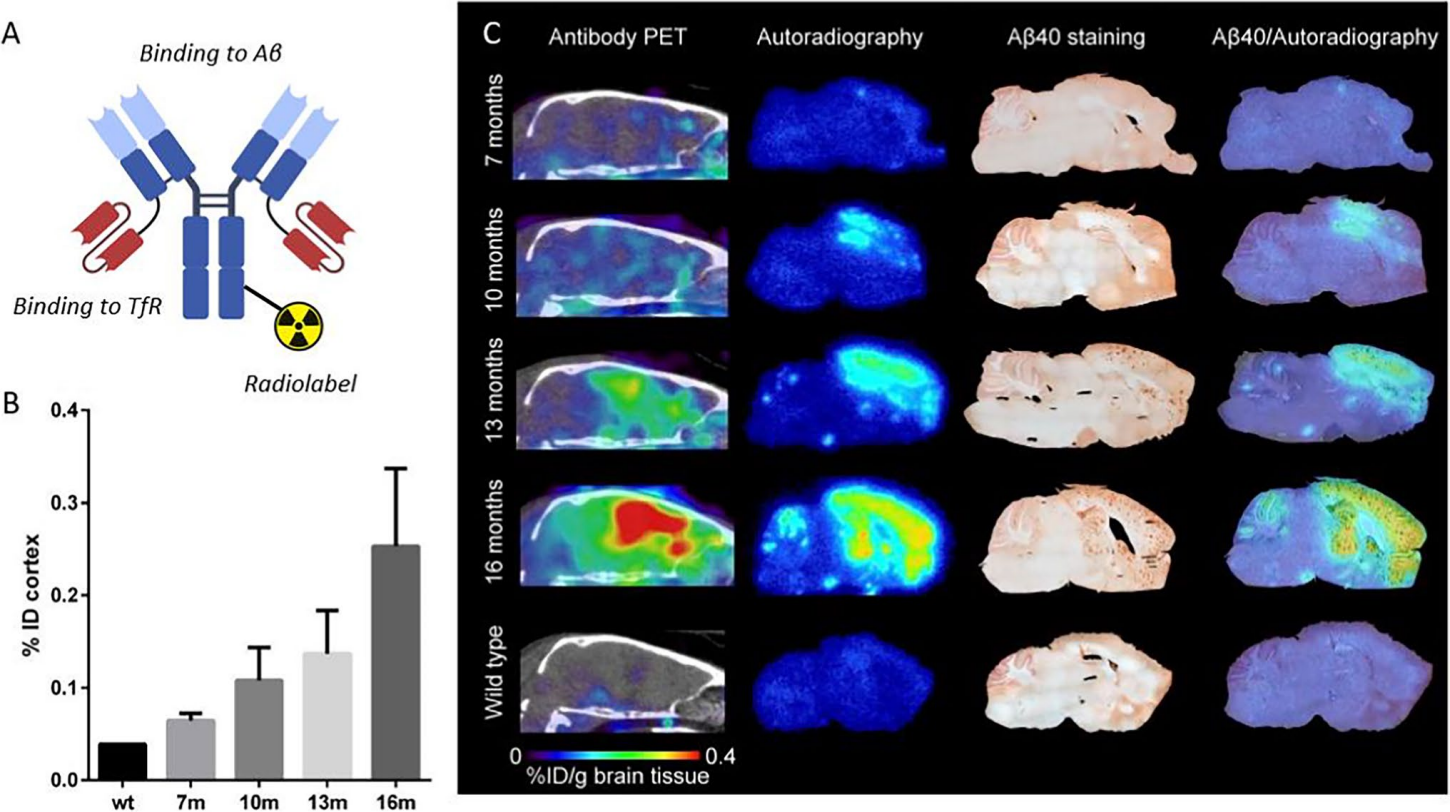
Imaging of amyloid (Aβ) with the bispecific antibody-based radioligand [^124^I]RmAb158-scFv8D3. (**A**) Schematic illustration of a bispecific antibody that binds to the transferrin receptor (TfR) for facilitated delivery across the blood–brain barrier and to Aβ aggregates in the brain. (**B**) Cortical [^124^I]RmAb158-scFv8D3 concentrations measured by PET and expressed as %ID/g at 6 days post injection in wild-type (wt) and tg-ArcSwe mice of different ages. (**C**) Sagittal PET images, ex vivo autoradiography of sections prepared post PET scanning, *in vitro* Aβ40 immunohistochemistry and an overlay of Aβ40 immunohistochemistry and autoradiography show pathology progression including the appearance of pathology in the cerebellum. Figure (**B**) and (**C**) obtained from Meier *et al*. 2018 with permission from the publisher (40).

Aggregation-prone Aβ42 dominates pathology in many of the frequently used animal models, including the knock-in *App*^*NL−G−F*^ model and the models that harbor a presenilin mutation, which favors production of Aβ42. The advantage of dominant Aβ42 pathology is that Aβ accumulation in the brain is faster, and thus, animals can be used at a younger age leading to reduced housing costs. However, the clear disadvantage is the difference to human sporadic AD where Aβ40 is the major species, and hence, translatability of mechanistic findings in animal models characterized by dominant Aβ42 pathology can be debated. Especially the morphology of the plaques appear to differ, where dominant Aβ42 pathology leads to smaller cores and potentially to more diffuse pathology that is less well detected by amyloid radioligands and is likely to require higher molar activity as discussed previously. A recent study in *App*^*NL−G−F*^ and APPPS1-21 models, representing limited and moderate fibrillary Aβ pathology respectively, showed that fibrillary Aβ contributed 16-fold more to the [^18^F]florbetaben signal than diffuse Aβ (81). Due to the much more abundant diffuse pathology in *App*^*NL−G−F*^, about 80% of the PET signal was still derived from diffuse pathology. However, the total signal was low, as knock-in *App*^*NL−G−F*^ mice at the age of 10 months showed only a 10% increase in brain retention of [^18^F]florbetaben compared to wild-type mice (21). This can be compared to the 100% higher PET signal in *App*^*NL−G−F*^ and tg-ArcSwe mice compared to wild-type mice already at the age of 7–8 months with the antibody-based radioligand [^124^I]RmAb158-scFv8D3 that is likely to detect mainly diffuse Aβ aggregates (Fig. 4) (20, 40).

Another aspect that must be considered when using Aβ models that are associated with fast accumulating pathology is the animal age; aging in itself may be important – is a young mouse comparable to an aged human?

Finally, analogous to human AD, the cerebellum in most animal models of Aβ pathology is spared from dense-core Aβ plaques longer than the rest of the brain. However, also this appears to differ between different models and is of importance when deciding on the definition of a “positive PET scan”. If extensive pathology is found in the cerebellum, SUVR using the cerebellum as a reference region may underestimate the level of pathology (77). Also here, antibody-based radioligands have revealed a wide-spread cerebellum pathology in some of the models (Fig. 4C) (20, 37). This could be of particular importance when using PET to evaluate the effect of Aβ reducing therapies.

### PET Studies of Drug Effects in Aβ Models

PET is increasingly used in clinical trials of new drug candidates aimed at reducing Aβ production or clearing brain Aβ. Thus, the rationale of using PET in Aβ models in preclinical drug development has also increased. This may be one of the most relevant applications of PET in animal models, as the main objective of such studies is to verify reduced Aβ levels in treated mice compared to non-treated, and at the same time, other aspects related to age and transgene effects may be less crucial. Nevertheless, if amyloid radioligands are used, the model must include Aβ aggregates that are relevant to AD and at the same time visible with PET, ideally at a relatively early age so that the study can be carried out over an extended time to make use of the possibility of PET to longitudinally follow individual animals.

**Fig. 5:**
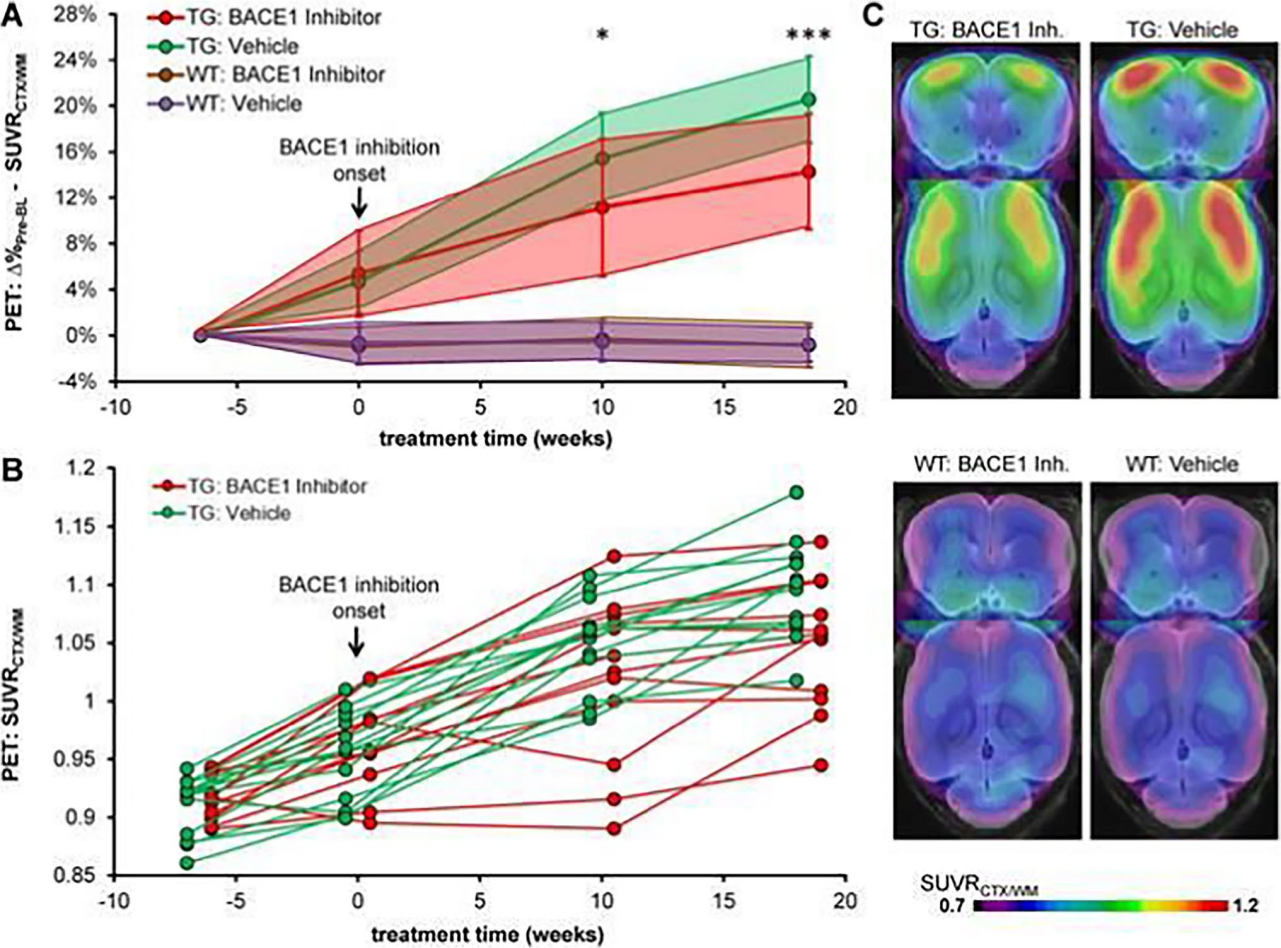
Longitudinal amyloid imaging of β-secretase inhibition. (**A**) Cortical [^18^F]florbetaben signal (mean ± standard deviation) relative to signal obtained in first baseline scan 6 weeks prior to start of vehicle or inhibitor treatment in transgenic (TG) and wild-type (WT) mice. (**B**) Individual progression of the cortical amyloid signal in transgenic mice. (**C**) Amyloid-PET signal intensities in the frontal cortex in mice during the terminal scan after 18.5 weeks of vehicle or inhibitor treatment. Coronal and axial slices illustrate group SUVR averages upon a T1 weighted MRI template. *p < 0.05; **p < 0.001. Figure from Brendel *et al*. 2018 with permission from the publisher (83).

Inhibitors and modulators of γ- or β-secretases belong to one class of drugs that have been studied with PET in animal models. Both γ- and β-secretases are involved in the production of Aβ from membrane bound *APP*, and thus, inhibition reduces brain Aβ. The first published large-scale longitudinal study described γ-secretase modulator RO5506284 treatment in APP_swe_ animals that were 12 months old when the study started (82). At 4 months into the treatment, PET imaging with [^18^F]florbetaben revealed a trend towards decreased SUVR_ctx/cer_ in treated mice compared to non-treated, and this trend became significant 2 months later, i.e. when mice were 18 months. An interesting finding was that RO5506284 treatment in animals with an increased baseline SUVR_ctx/cer_, indicating a higher brain amyloid burden at the start of the study, was less effective than in animals with lower baseline SUVR_ctx/cer_. In fact, treated animals with a high baseline SUVR_ctx/cer_ displayed higher SUVR_ctx/cer_ after 6 months of treatment compared to non-treated animals that entered the study with low baseline SUVR_ctx/cer_. This implies that studies of drug effects should be carried out in longitudinal designs to enable each individual to act as its own control, rather than cross-sectional designs in which group averages are compared. The same researchers then went on to further refine the protocol by including two baseline scans, separated by six weeks, to estimate the “natural Aβ deposition rate” in different brain regions prior to treatment (Fig. 5) (83). In line with their previous study, animals with low amyloid load at baseline were more efficiently treated with the β-secretase inhibitor RO5508887 at a daily dose of 100 mg/kg. Further, the treatment completely stopped Aβ accumulation in regions with low accumulation rates while regions with high accumulation rates were less well-treated. In another study, β-secretase inhibitor JNJ-49156981 was investigated over 10 months in APPPS1-21 mice that were only 6–7 weeks old at the start of the study (84). PET imaging with [^18^F]florbetapir revealed a small but significant treatment effect. However, the study also reported age-dependent alterations in radioligand binding in wild-type mice along with high non-specific binding that somewhat compromised the interpretation of the study.

Since all of the above-mentioned investigations were initiated in young animals with fairly low levels of pathology, they primarily show reduced de novo formation of Aβ plaques, rather than clearance of Aβ plaques (82–84). Similar results were also obtained in a PET study using the antibody-based PET radioligand [^124^I]RmAb158-scFv8D3 for detecting effects of β-secretase inhibitor NB-360 in tg-ArcSwe mice (40). Compared to the previous studies with amyloid radioligands [^18^F]florbetaben and [^18^F]florbetapir, a larger difference between treated and non-treated animals was observed with [^124^I]RmAb158-scFv8D3. The better differentiation with [^124^I]RmAb158-scFv8D3 was probably due to the detection of diffuse Aβ aggregates, which are likely more accessible to treatment, compared to the dense core of amyloid plaques (40). The antibody-based radioligand and [^11^C]PIB was then used to monitor effects of β-secretase inhibition in older tg-ArcSwe and *App*^*NL−G−F*^ mice that displayed advanced pathology already at the start of the treatment (20). Interestingly, the antibody-based radioligand [^124^I]RmAb158-scFv8D3, but not [^11^C]PIB, was able to detect reduced brain Aβ levels in the brain. Thus, in line with the previous studies, amyloid imaging with [^11^C]PIB indicated that treatment was not effective in mice with high baseline pathology (which was the case for all mice in the present study as it included old mice only). However, the antibody-based radioligand that shows a preference in binding to diffuse aggregates, which may represent a more dynamic pool of Aβ, including de novo formed aggregates, did indicate a treatment effect (20). The different readouts with the two radioligands highlight the complexity of Aβ pathology and the many different forms of it, and further, the importance of careful study design and selection of radioligands. Another aspect to keep in mind when performing PET studies in old animals to evaluate treatment effects is that cerebellum pathology may be present at the start of the study. The use of cerebellum as a reference region to calculate SUVR may underestimate the effect of the treatment. SUVR will increase if pathology in the cerebellum, which is likely to be less mature and more diffuse, is easier to abolish with treatment than the more mature pathology in the rest of the brain. For example, NB-360 effectively reduced Aβ in the cerebellum in tg-ArcSwe and *App*^*NL−G−F*^ mice (20). Consequently, if SUV decreases in the cerebellum, it will increase the SUVR_ROI/cer_, and this increase may be interpreted as a lack of effect. Thus, it is essential to investigate both SUV and SUVR, or potentially perform full pharmacokinetic modelling based on radioligand concentrations in blood.

In addition to secretase inhibitors, a few other therapy strategies have also been evaluated with preclinical PET with varying success. In one study, 16–17 month old APP23 mice were imaged with [^11^C]PIB PET before and after treatment with liposomes functionalized with a phosphatidic acid that had previously been described to inhibit Aβ aggregation and promote brain Aβ elimination (85). The effectiveness of the drug itself was questionable as post mortem immunohistochemistry could not confirm any effect on brain Aβ levels. However, in line with previously described secretase inhibitor studies, large inter-animal variability in the baseline scan showed that studies carried out also in old mice should be designed so that the effect can be estimated in each individual mouse, rather than at group level, or alternatively, the study must include a large number of animals in each treatment group.

Anti-Aβ antibodies have also been studied in animal models with PET. A therapeutic dose of RmAb158 as well as of a tenfold lower dose of the bispecific RmAb158-scFv8D3 that was engineered for facilitated brain delivery were given to 18 month old tg-ArcSwe mice (86). Animals treated with RmAb158 showed a 20–25% decrease in SUVR_ctx/cer_ when imaged with antibody-based radioligand [^124^I]RmAb158-scFv8D3, while the decrease was double in animals treated with brain-penetrant RmAb158-scFv8D3. Since the PET scans were conducted after a single injection of the therapeutic antibodies, which did not significantly alter total levels of brain Aβ, the decreased SUVR_ctx/cer_ is likely to reflect target engagement, and thus blocking of binding sites for the radioligand that was based on the same antibody, rather than reduced brain Aβ levels. Another study compared brain retention of [^125^I]RmAb158 and [^125^I]RmAb158-scFv8D3 with single-photon emission computed tomography (SPECT) imaging over a period of four weeks, and showed that the bispecific antibody was present in the brain at a higher concentration than [^125^I]RmAb158 at all times despite lower blood concentrations (87). A related study of [^125^I]3D6 showed a general low brain distribution with the exception of intense “hot-spot” accumulation, potentially a consequence of local antibody interaction with CAA (88). PET imaging of target engagement may be important evidence and an aid in dose selection prior to longer treatment studies.

### Future

The logistic and cost benefits of using ^18^F-amyloid radioligands instead of [^11^C]PIB in preclinical models are central. However, as discussed above, the increased non-specific binding may reduce the potential of ^18^F-amyloid radioligands in animals with lower levels of pathology. Thus, the development of ^18^F-amyloid radioligands with improved specific to non-specific signal could increase the use of preclinical PET as a tool to investigate drug effects on Aβ pathology. There is already today one ^18^F-amyloid radioligand, [^18^F]flutafuranol (^18^F-AZD4694, [^18^F]NAV4694, Fig. 2) with lower white matter distribution compared to the FDA-approved ^18^F-amyloid radioligands (89, 90). Until today, [^18^F]flutafuranol has been less available to preclinical PET-groups and thus used sparsely, although it has been shown to readily visualize amyloid in the McGiIll-R-Thys-APP rat model (64).

Antibodies and other protein-based radioligands have been described as a novel class of radioligands for PET imaging of Aβ (91). Although their slow pharmacokinetics is a clear draw-back, their ability to detect Aβ beyond amyloid is interesting. Smaller bispecific formats appear to show more favorable and PET compatible pharmacokinetics than large antibody-like formats (35, 92, 93). Further, bispecific antibodies have also been labelled with the clinically preferred radionuclide ^18^F (94). Luminescent conjugated oligothiophenes (LCOs) and polythiophenes (LCPs), which have been used to image and distinguish between different forms of Aβ aggregates on tissue sections as well as *in vivo* with multiphoton microscopy, are interesting scaffolds for the development of new specific radioligands for imaging of Aβ, including non-amyloid Aβ (95, 96). Radioligands that image non-amyloid components of Aβ are of interest especially for evaluation of the emerging AD immunotherapies, e.g. *aducanumab* and l*ecanemab*, were at least the later antibody was raised against soluble oligomers.

In addition to Aβ, PET imaging of targets related to parallel and sequential pathological changes in the brain are attracting more attention in preclinical AD research. Brain metabolism studied with [^18^F]FDG, a glucose analogue, has been used in several studies in Aβ models, often in combination with amyloid radioligands (16, 19, 97–100). The importance of neuroinflammation in AD has gained increased attention during the past decade. Several radioligands have been developed for imaging of the 18-kDa translocator protein (TSPO), which is highly expressed by activated microglia (101). Radioligands visualizing TSPO have therefore also been used in AD models, and often together with an amyloid radioligand or [^18^F]FDG (100, 102, 103). These radioligands suffer from somewhat low specific binding, and in addition, TSPO is not expressed solely by microglia but also found in astrocytes and endothelial cells. Monoamine oxidase B, expressed by reactive astrocytes, is another target related to neuroinflammation, and levels have been shown to increase with Aβ pathology in mouse models (104, 105). However, as for TSPO, monoamine oxidase B is not a specific marker for inflammation. Many new targets are therefore investigated as potential imaging markers for neuroinflammation. One of these, that is especially interesting for AD applications, is the triggering receptor expressed on myeloid cells 2 (TREM2) found in microglia. Loss of function mutations have been shown to increase AD risk in humans, and further, TREM2 levels appear to be increased in the presence of Aβ pathology (106). TREM2 is a target under investigation for therapeutic antibodies, and could therefore also be interesting from an imaging perspective (107). Another class of radioligands that has emerged during the last 4–5 years bind to the synaptic vesicle protein 2A (SV2A) (65). SV2A is a presynaptic protein involved in neurotransmitter release and storage. In PET, it is used as a proxy for the number of functional synapses, and thus, to estimate synaptic density. A reduced SV2A PET is interpreted as a sign of neurodegeneration. Although the radioligand was used in human AD patients soon after its first description, several animal studies have been published later. However, results are not clear-cut as the difference between the signal in Aβ models and healthy wild-type mice seem to be rather modest (43, 108, 109). It is likely that radioligands for imaging of novel targets related to neuroinflammation and synaptic changes will be used more frequently either on their own or in multi-radioligand designs, e.g. in combination with Aβ-PET, in preclinical effect validation of anti-Aβ drugs.

## SUMMARY AND CONCLUSION

Preclinical PET imaging in animal models of Aβ pathology allows for *in vivo* effect monitoring of anti-Aβ treatments and may as such be an important aid in preclinical drug development. However, the selection of animal model and radioligand is crucial. Large inter-animal variability has been shown for a number of models, and at all stages during pathology progression. Variability, that recapitulates the situation in human AD patients, poses a challenge for cross-sectional study designs. This is especially true for today’s amyloid radioligands that display a rather weak signal in many models due to the lack of large dense-cored plaques which in turn leads to a small difference between genetically modified mice and their wild-type controls, and less sensitive detection of alterations in brain Aβ levels due to treatment. Further, it appears as [^11^C]PIB may be advantageous compared to ^18^F-radioligands such as [^18^F]florbetaben, [^18^F]flutemetamol and [^18^F]florbetapir due to less non-specific binding. However, ^11^C is not ideal for multiple scans per radioligand synthesis due to its short half-life and therefore, the cost of a well-powered preclinical [^11^C]PIB PET study will increase. Antibody-based radioligands may be more sensitive than amyloid radioligands and can be used to detect Aβ aggregates beyond plaques. However, the possibility to translate these radioligands to human use is unclear, although some efforts with smaller ^18^F-labelled variants have been described. Despite this, they may be useful research tools as they can be made to match emerging immunotherapeutic antibodies, and can as such be used to show target engagement of the therapeutic antibody. The prospect of *in vivo* determination of which forms of Aβ aggregates that are affected and altered by treatment and disease progression is likely to be essential in the development of new efficient therapies for AD.
